# The role of liver cancer stem cells in hepatocellular carcinoma metastasis

**DOI:** 10.1080/15384047.2024.2321768

**Published:** 2024-02-23

**Authors:** Qinghui Niu, Susu Ye, Liu Zhao, Yanzhi Qian, Fengchao Liu

**Affiliations:** a Liver Disease Center, The Affiliated Hospital of Qingdao University, Qingdao, China; b School Hospital, Qingdao University of Science and Technology, Qingdao, China

**Keywords:** HCC, cancer stem cells, metastasis, metastatic cascade, CSCs niche

## Abstract

Metastasis accounts for the vast majority of cancer deaths; however, this complex process has yet to be fully explained. To form metastases, cancer cells must undergo a series of steps, known as the “Metastatic cascade”, each of which requires a specific functional transformation. Cancer stem cells (CSCs) play a vital role in tumor metastasis, but their dynamic behavior and regulatory mechanisms have not been fully elucidated. Based on the “Metastatic cascade” theory, this review summarizes the effect of liver CSCs on the metastatic biological programs that underlie the dissemination and metastatic growth of cancer cells. Liver CSCs have the capacity to initiate distant organ metastasis via EMT, and the microenvironment transformation that supports the ability of these cells to disseminate, evade immune surveillance, dormancy, and regenerate metastasis. Understanding the heterogeneity and traits of liver CSCs in these processes is critical for developing strategies to prevent and treat metastasis of advanced hepatocellular carcinoma (HCC).

## Introduction

HCC is the sixth most common cancer and the third most common cause of cancer-related death.^1^ Surgical therapy is the primary treatment for patients with solitary tumors at an early stage. For these patients, resection is associated with 5-year survival exceeding 60%; however, up to 70% of these patients suffer tumor recurrence by 5 years.^2^ Because most HCCs occur within a background of liver disease and cirrhosis, liver transplantation is often the optimal surgical treatment option for HCC patients with early stage tumors and accounts for approximately 20–40% of all liver transplantation recipients worldwide.^3^ As reported previously, the recurrence of HCC after liver transplantation ranges between 10 and 20% and may be higher as the selection criteria are expanded.^4^ Tumor recurrence after liver transplantation is frequently associated with extrahepatic metastasis, particularly in the lungs and bones.^3,5,6^ The poor prognosis of HCC remains a great challenge principally due to its high rate of metastasis. Therefore, a deeper understanding of the mechanisms underlying HCC metastasis is urgently required.

The development of metastases requires a variety of cellular mechanisms termed the ‘metastatic cascade’, which includes acquiring invasive potential, developing the capability to disseminate from their primary site, promoting vascular invasion, surviving while circulating in the bloodstream, entering and exiting dormancy, establishing new cellular surroundings in secondary sites, stimulating angiogenesis, and evading the host immune system.^7–9^ Tumor metastasis is a complex process that takes several years to achieve. Only a small fraction of the cells can develop the capability to metastasize to distant sites. These cells must undergo a complex transformation acquiring a variety of phenotypic traits to survive various stresses during the process and become proficient at initiating metastatic disease outgrowth. This specialized subset of cells responsible for tumor metastasis remains largely incurable. An increasing number of studies have suggested that metastatic colonization of distant organs is initiated by cancer cells with stem cell – like phenotype.^10–13^

Cancer stem cells (CSCs) are a small subpopulation of stem-like tumor cells capable of self-renewal, differentiation, and tumorigenesis.^14^ There has been a marked increase in research focused on the identification and characterization of CSCs in general, and specifically liver CSCs in the past two decades, which has brought new hope to the diagnosis and treatment of HCC. Several liver CSC markers have been identified, including CD133, CD90, EpCAM, CD13, CD44, OV6, CD24, α2δ1, ICAM-1, Lgr5, and K19.^15,16^ Although the mechanisms remain unclear, an increasing number of studies have confirmed the link between liver CSCs and HCC metastasis.^17,18^ Understanding the role of liver CSCs in the development of HCC cell metastasis could provide new insights into the diagnosis and treatment of metastatic HCC. In this review, numerous studies that have addressed the involvement of liver CSCs in each step of the metastatic process have been examined to further explore the important role of liver CSCs in HCC metastasis and thereby provide ideas for better treatment of HCC metastasis.

## Interplay of liver CSCs and EMT in HCC

To investigate and comprehend the molecular mechanisms of tumor metastasis inevitably brings epithelial – mesenchymal transition (EMT) into the picture. EMT is a cellular process by which epithelial cells lose their epithelial characteristics and acquire the phenotype of mesenchymal stem cells. In cancers, aberrant activation of this transformation process can be disastrous, as the differentiated epithelial cells are endowed with migratory and invasive characteristics, thereby initiating the dissemination of tumor metastasis.^19^ The scope of biological processes that have been found to be driven by EMT extends far beyond the initial steps of cancer cell invasion. Accumulating evidence indicates that experimental activation of EMT confers stemness properties, such as CSCs, on epithelial tumor cells.^20,21^

An accumulating body of research indicates that EMT in HCC cells is always accompanied by the acquisition of stemness.^22–28^ Although the full relationship between EMT and HCC stem cells remains unclear, it is widely recognized that TGF-β1-induced EMT promotes tumor cell transformation into the CSC phenotype, including HCC cells.^29^ HIF‐1α reportedly induced the EMT process of transforming HCC cells into cells with CSC characteristics, which was mediated by Notch1 activation.^30^ Interestingly, not only does induction of EMT promote stemness, conversely, induction of stemness also promotes the EMT process. MHCC97-L cells were empowered with CSCs properties by ectopic co-expression of stemness-related genes Oct4 and Nanog and underwent EMT to promote tumor migration and invasion/metastasis in vitro and in vivo.^31^ Another study reported that CSC spheres, induced by culturing in a unique medium containing neural survival factor-1, possessed an increased metastatic potential which promoted the EMT process.^32^ Nonetheless, certain experimental evidence does not support a correlation between EMT and stemness. For example, Vimentin+CD133- HCC cells demonstrate EMT phenotypes whereas Vimentin-CD133+ stem cells do not have EMT phenotypes, and Vimentin-CD133- cells exhibit more aggressive metastasis than vimentin-CD133+ cells.^33^ Therefore, the correlation between HCC stem cells and the EMT requires further investigation.

## Liver CSCs niche required for CSCs maintenance and survival

**Figure 1.**
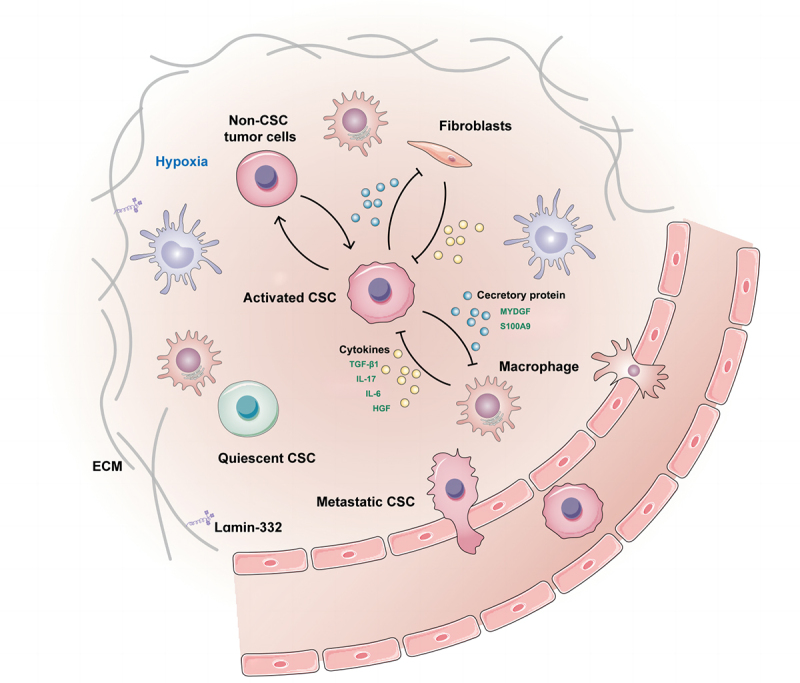
Liver CSCs within the niche.

The tumor microenvironment (TME) surrounding tumors communicates with tumor cells, leading to the growth, invasion, and metastasis of various tumors, including HCC. It has long been recognized that interactions between cancer cells, endothelial cells, stromal fibroblasts, and immune cells are mediated by a number of cytokines or growth factors, as well as alterations in tissue oxygen tension, and that the architecture of the adjacent extracellular matrix (ECM) profoundly impacts tumor progression^34^ (Figure 1). Mounting evidence has demonstrated the presence and role of environmental inflammatory factors such as TGF-β1,^29^ chemokine,^35^ IL-17,^36^ lipopolysaccharide,^37^ IL-6,^38^ and HGF^39^ in the HCC TME in the regulation of non-CSC-acquired stemness or CSC-maintained stemness (Figure 1). Under the stimulation of the inflammatory factor IL-6, normal liver stem cells may transform into metastatic liver CSCs, thereby promoting the metastasis of liver cancer.^40^

There is a close crosstalk between liver CSCs and niche cells, which not only maintains its own stemness, but also sows the “seeds” for tumor metastasis.

Distinct regions within the microenvironment harboring CSCs are termed the “CSC niche”. As shown in several tumors, interactions between CSCs and their niches are critical for metastatic progression.^41^ Laminin-332, an extracellular matrix protein complex, is part of the specialized liver CSCs niche in maintaining cell stemness, which leads to chemoresistance and quiescence.^42^ An hypoxic microenvironment is common in solid cancers, particularly in HCC, and is an important component of the stem cell niche that promotes the stemness of HCC via HIF-1α.^43–45^ Hypoxia is sufficient to promote invasion and accelerate metastatic outgrowth, which is associated with rapid dedifferentiation of tumor cells into liver CSCs.^46^ Liver CSCs can construct their niche by secreting proteins that communicate with macrophages. It has been reported that the secretory protein MYDGF released by HCC cells under hypoxic conditions might be involved in the enhanced self-renewal of liver CSCs. Furthermore, MYDGF was also found to induce chemotaxis of macrophages into tumor tissue then release cytokines, which ultimately amplified inflammation of the tumor microenvironment and accelerated HCC progression.^47^ Secretory protein S100A9 secreted from tumor-associated macrophages may enhance HCC cells to acquire stemness through paracrine signaling.^48^ Liver CSCs also facilitate migration of macrophages by upregulating chemokine-related genes and elevating metastatic potential by recruiting macrophages in vivo.^49^ Cancer-associated fibroblasts, a component of the stromal fraction in the TME, contribute to the stemness of liver CSCs by secreting growth factors.^50,51^ Lymphatic endothelial cells are also important components of the liver CSCs niche microenvironment, and the interaction between liver CSCs and lymphatic endothelial cells promotes the self-renewal and immune escape of hepatoma stem cells via activation of IL-17A signaling.^52^ From these studies, it is evident that there is close crosstalk between liver CSCs and niche cells, which not only maintains their own stemness but also sows the “seeds” for tumor metastasis (Figure 1).

## Effect of liver CSCs-mediated ECM remodeling on metastasis of HCC

The extracellular matrix (ECM) undergoes dramatic changes during cancer progression and plays a central role in the metastatic process.^53^ The proliferation, invasion, and dissemination of tumor cells are accompanied by dramatic changes in the mechanical properties (stiffness) of the ECM in the cancer cell niche.^54^ One study provided evidence that tumor tissues from metastatic HCC patients possessed a higher matrix stiffness than the non-metastatic group.^55^ Multiple tumor studies have shown that diverse matrix stiffnesses can contribute to the self-renewal of CSCs. In HCC, the link between CSCs stemness and extracellular matrix stiffness remains controversial. Soft matrix stiffness promotes the acquisition of stemness in HCC cells.^54,56^ A soft spot matrix created by CD133+ liver CSCs through ECM remodeling could enhance stemness maintenance, drug resistance, and metastatic dissemination of HCC cells.^57^ However, higher matrix stiffness was reported to promote the CSC phenotype and reduce sorafenib-induced apoptosis.^58^ Recently, Li et al. showed that a stiffer matrix significantly potentiated LCSC stemness compared to a soft matrix by establishing a three-dimensional hydrogel for culturing liver CSCs.^59^ Another recent study reported that α2δ1+ liver CSCs created a stiff ECM through the secretion of lysyl oxidase (LOX), which was subsequently required for the acquisition and maintenance of HCC CSC characteristics.^60^ These results would support the postulate that dynamic alterations in the stiffness of the liver CSCs niche could be responsible for regulating CSC self-renewal and phenotypes throughout the natural history of HCC metastasis.

## Relationship between liver CSCs and circulating tumor cells

Circulating tumor cells (CTCs) are malignant cells that extravasate into the blood vessels (intravasation) from primary tumors and are considered the “seeds” of distant metastasis.^61^ Many previous studies have demonstrated that CTCs possess the CSC phenotype.^62–64^ However, the characteristics of circulating CSCs in HCC are poorly understood. In HCC, 71.4% of patients had CTCs positive for the CSC marker CD44; thus, they had a significant population of CTCs with CSC properties.^65^ Most current CTC capture methods are based on epithelial cell adhesion molecule (EpCAM) detection, including HCC.^66,67^ EpCAM is also a commonly used biomarker for liver CSC isolation.^18,68^ Stem cell – like phenotypes have been confirmed in HCC EpCAM+ CTCs by testing the expression of stem cell-related markers and high tumorigenic ability.^69^

Numerous studies have found that the circulating CSC count has significant importance in monitoring and prognosis of patients with HCC by detecting liver CSCs in the blood via a variety of surface markers. CD45-CD90+ CSCs were detectable in 90% of HCC patients, and the number of CD45-CD90+ cells in the tumor tissues was significantly positively correlated with the number of CD45-CD90+ cells in the blood samples.^70^ Detection of circulating CSCs (CD45− CD90+ CD44+cells) is highly accurate in predicting HCC recurrence following hepatectomy.^71^ Utilizing ICAM-1 as a liver CSC marker, Liu et al. discovered that ICAM-1-positive CTCs were detected in 50% of all cases, and that these cells correlated with worse clinical outcomes.^72^ Guo et al. found that CTCs with stem-like phenotypes, rather than the entire population of CTCs, might reflect the micrometastatic status, which could not be detected by routine imaging techniques, and enable a more accurate estimate of recurrence risk of HCC patients.^73^

## Quiescence/Dormancy liver CSCs in HCC metastasis

HCC metastasis typically becomes clinically evident years following the removal of a primary tumor by resection or liver transplantation, derived from a small number of disseminated cancer cells that survive as latency-competent cancer (LCC) cells. LCC cells show stem cell-like characteristics that are essential for their survival under immune surveillance, ultimately developing metastatic outgrowth under permissive conditions.^74^ CSCs, through their immunomodulating features by secreting a variety of factors or cytokines, evade immune surveillance and immune attack, persisting in the form of quiescence and dormancy.^75^ Thus, CSCs cannot be detected by the immune system before the development of metastasis and recurrence, due to the progression of dormancy.

Understanding the biological properties of dormant CSCs and their reactivation mechanisms is essential for developing therapeutic approaches to prevent cancer recurrence and metastasis. It has been demonstrated that CD13 is a semiquiescent CSC marker in human liver cancer cell lines and clinical samples. CD13 protects cells from apoptosis after genotoxic chemo/radiation stress by causing the cells to predominate in the G0 phase of the cell cycle and reducing ROS-induced DNA damage.^76^ Another study showed that CD13+ liver CSCs can sustain quiescence and resistance to chemotherapeutic agents via aerobic metabolism of tyrosine.^77^ Side population (SP) CSCs are surrounded by a specialized CSC niche rich in laminin-332, which maintains liver CSCs in a quiescent state.^42^

## The effect of liver CSCs in immune evasion

In HCC, immune evasion is established early and is a progressive and continual process that peaks at intermediate stage II tumors.^78^ Cancer cells, dissociated from the immunosuppressive microenvironment of the primary tumor, become vulnerable to immune surveillance which requires escape from immune-mediated elimination if they are to form metastases.^79^ CSC immune evasion is key to maintaining the tumorigenic process of CSCs by changing their own molecular expression and reprogramming the immune response.^80,81^ Natural killer (NK) cells have been demonstrated to play critical roles in the first line of immunological defense against cancer development, including HCC.^82^ EpCAM+ liver CSCs have been shown to be resistant to NK cell-mediated cytotoxicity by upregulating CEACAM1 expression.^83^ CD133+ liver CSCs show a strong immune escape ability by interacting with lymphatic endothelial cells and activating IL-17A signaling.^52^ One study reported that liver CSCs can evade the adaptive immune response through activated regulatory CD4+/CD25+/FoxP3+ T cells (Tregs) in a paracrine manner.^84^ The mechanism of liver CSCs immune evasion requires further investigation.

## Metabolism reprogramming is crucial to maintain liver CSC stemness

Metabolic reprogramming is considered a major hallmark of tumorigenesis. This reprogramming provides supplementary energy, cellular building blocks, and redox deficits for their eventual survival and metastases.^85,86^ CSCs exhibit altered metabolism and energy balance to meet a hostile TME for survival and stemness maintenance.^87,88^ In HCC, cells have been shown to retrodifferentiate to CSCs, accompanied by metabolic reprogramming, including changes in mitochondrial activity with reduced membrane potential, low ATP production, and high lactate production.^89^ Wei et al. demonstrated that liver CSCs regulate stemness properties by regulating the mitochondrial respiratory function.^90^

Glucose metabolism is essential for generating sufficient energy to maintain complex tissues, including tumors. Cancer cells must undergo critical changes to meet their altered energy needs by decoupling glycolysis from pyruvate oxidation, thereby increasing the glycolysis pathway despite the availability of sufficient oxygen, a process known as the Warburg effect.^91^ Studies have shown that glucose metabolism reprogramming, including upregulation of glycolysis,^92–94^ increased oxidative phosphorylation (OXPHOS),^95^ and elevated glucose uptake^96^ regulate the stemness of liver CSCs.

Reprogramming of amino acid metabolism is also crucial for maintaining CSC stemness. Chemoresistant HCC cells were found to exhibit a CSC phenotype with altered metabolism in a metabolically quiescent state with glucose independence and relied on glutamine metabolism compared to chemo-sensitive HCC cells.^97^ Similarly, glutamine metabolism has been confirmed to play an important role in liver CSC maintenance, and that targeting glutamine metabolism or glutaminase 1 (GLS1) could suppress CSC traits in HCC.^98^ These studies indicated that the survival and self-renewal of liver CSCs depended on glutamine. However, a recent study found that liver CSCs could maintain their stemness characteristics even upon glutamine deprivation.^99^ CD13+ liver CSCs were reported to be dependent on the aerobic metabolism of tyrosine rather than glucose as an energy source, and tyrosine metabolism could also generate nuclear acetyl-CoA to acetylate and stabilize Foxd3, thereby allowing CD13+ CSCs cells to sustain quiescence and resistance to chemotherapeutic agents.^77^ Alanine-glyoxylate aminotransferase (AGXT) has been demonstrated to regulate the metabolic processes of serine, glycine, and alanine, and one study implied that the metabolic processes of serine, glycine, and alanine regulated by AGXT were crucial for liver CSC stemness.^100^

Lipid metabolism appears to be a critical property of liver CSCs. Fatty acid metabolism is the core of lipid status harmonization in lipid metabolism. In malignant cells, carbon is characteristically diverted from energy production to fatty acids for biosynthesis of membranes and signaling molecules. Fatty acid oxidation (FAO)-mediated energy generation is critical to cancer cell survival and metastasis, and ectopic activation of FAO maintains CSCs under conditions of glycolytic deficiency.^101^ It has been reported that activation of FAO and inhibition of oxidative phosphorylation and ROS production play an important role in the maintenance of liver CSCs stemness.^102^ Another study showed that an increase in FAO activity facilitates the maintenance and self-renewal of liver CSCs.^103^

## Liver CSCs promote angiogenesis

Solid tumors require new blood vessels for their growth and metastasis. Considerable evidence has characterized links between CSCs and tumor angiogenesis in the surrounding microenvironment, which are directly associated with cancer development and metastasis. Studies have shown that CSCs from several cancers, such as glioblastoma,^104^ ovarian cancer,^105^ lung cancer^106^ and breast cancer,^107^ can transdifferentiate into endothelial cells and further form functional neovasculature. Yang et al. determined that high expression levels of liver CSC biomarkers were related to tumor angiogenesis and poor prognosis of HCC.^108^ It was previously demonstrated that cancer stem-like sphere cells from HCC cells were able to differentiate into endothelial cells, and this process was independent of VEGF and NOTCH signaling, but dependent on the activation of Akt and IKK.^109^ Conigliaro et al. demonstrated that CD90+ liver CSCs could affect endothelial cells and promote angiogenesis in a pro-metastatic manner by releasing exosomes containing H19 lncRNA.^110^ CD133+ liver CSCs have also been shown to promote tumor angiogenesis through neurotensin/interleukin-8/CXCL1 signaling.^111^ The stem cell-associated transcription factor Oct4 was recently shown to regulate the transition of CSCs to tumor endothelial-like cells in human liver cancer.^112^ These studies suggest that liver CSCs predominantly promote angiogenesis through paracrine effects or transdifferentiation into endothelial cells, which may contribute to the development of recurrence, metastasis, and angiogenic treatment resistance.

## Conclusions

**Figure 2.**
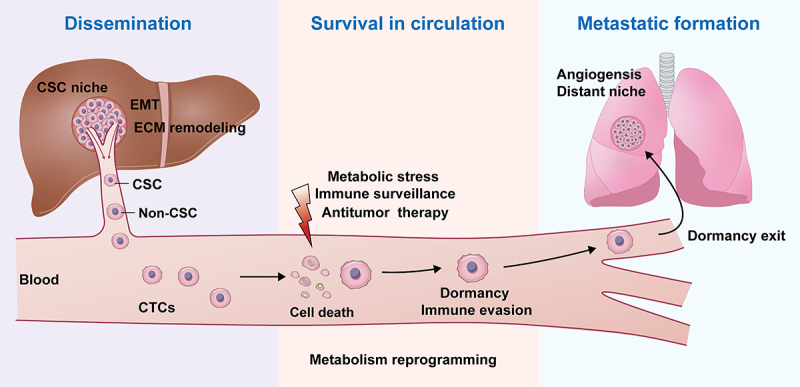
Mapping of liver CSCs in HCC metastatic cascade.

Metastasis is a complex tumor process that is the most lethal characteristic of cancer. Although usually not appearing clinically for years after initial discovery of a primary cancer, the presence of a tiny fraction of the malignant cells which are treatment-resistant, phenotypically CSCs, have been identified as responsible for tumor metastasis. Therefore, there is a need for more effective therapies that can effectively eradicate these residual cells. However, the development of effective treatments for metastases depends on the understanding of the mechanisms underlying the metastatic process from start to finish. Not all cells in a tumor can migrate from the primary site and metastasize. Only the relatively few cells circulating in the blood have been implicated, CSCs. An increasing number of studies have revealed that liver CSCs play an important role in various processes of HCC metastasis, including cell dissemination into the blood, adaptation to harsh environments, and construction of metastatic foci (Figure 2). As such, liver CSCs could become metastasis-initiating cells (MICs) by several cellular processes, including EMT, CSC niche interactions, ECM remodeling, then intravasate into the blood vessels, retaining their original stem cell phenotype. Liver CSCs can survive in the blood or other harsh environments through dormancy, sustained quiescence, and metabolic reprogramming. Liver CSCs play an important role in angiogenesis which enables the formation of metastatic lesions. In this review we have summarized the role of liver CSCs throughout the biological programs of the metastatic cascade. Understanding the role of liver CSCs in regulating HCC metastasis provides the critical foundation from which innovative new approaches and therapies can be developed to prevent or treat metastatic HCC.

liver CSCs play an important role in processes of HCC metastasis, including cell dissemination into the blood, survival in circulation, and metastatic foci formation.
